# Determination of Ultra-trace Rhodium in Water Samples by Graphite Furnace Atomic Absorption Spectrometry after Cloud Point Extraction Using 2-(5-Iodo-2-Pyridylazo)-5-Dimethylaminoaniline as a Chelating Agent

**DOI:** 10.3390/molecules22040487

**Published:** 2017-03-24

**Authors:** Quan Han, Yanyan Huo, Jiangyan Wu, Yaping He, Xiaohui Yang, Longhu Yang

**Affiliations:** 1 School of Chemical Engineering, Xi′an University, Xi′an 710065, China; huoyeye@163.com (Y.H.); hejin04c@126.com (Y.H.); yangxh1127@aliyun.com (X.Y.); 2 School of Chemistry and Chemical Engineering, Yan’an Universty, Yan’an 716000, China; wjy143@163.com (J.W.); longer666111@163.com (L.Y.)

**Keywords:** 2-(5-iodo-2-pyridylazo)-5-dimethylaminoaniline, rhodium, TritonX-114, cloud point extraction, graphite furnace atomic absorption spectrometry

## Abstract

A highly sensitive method based on cloud point extraction (CPE) separation/preconcentration and graphite furnace atomic absorption spectrometry (GFAAS) detection has been developed for the determination of ultra-trace amounts of rhodium in water samples. A new reagent, 2-(5-iodo-2-pyridylazo)-5-dimethylaminoaniline (5-I-PADMA), was used as the chelating agent and the nonionic surfactant TritonX-114 was chosen as extractant. In a HAc-NaAc buffer solution at pH 5.5, Rh(III) reacts with 5-I-PADMA to form a stable chelate by heating in a boiling water bath for 10 min. Subsequently, the chelate is extracted into the surfactant phase and separated from bulk water. The factors affecting CPE were investigated. Under the optimized conditions, the calibration graph was linear in the range of 0.1–6.0 ng/mL, the detection limit was 0.023 ng/mL for rhodium and relative standard deviation was 3.67% (*c* = 1.0 ng/mL, *n* = 11)．The method has been applied to the determination of trace rhodium in water samples with satisfactory results

## 1. Introduction

As a precious element of the platinum group elements (PGEs), rhodium is widely used in catalysts in the chemical and automotive industries due to its excellent catalytic activity. More than 80% of the world production of rhodium in the last decade was used in the production of automobile catalysts [1]. As a component in the typically used three-way catalysts, rhodium is indeed effective in reducing NO_x_ emissions, but as a result more and more rhodium is being released into the environment during the operation of converters [2]. Hence, a reliable and efficient analytical technique for studying and evaluating its future risk to human health and the ecosystem is required. As rhodium often occurs in environmental samples in complex matrices at low concentrations, its analysis often requires a highly sensitive analytical method with a pre-concentration step.

Several sensitive techniques such as graphite furnace atomic absorption spectrometry (GFAAS) [3,4], cathodic stripping voltammetry (CSV) [5,6,7], neutron activation analysis (NAA) [8,9], inductively coupled plasma-atomic emission spectrometry (ICP-AES) [10,11], and inductively coupled plasma-mass spectrometry (ICP-MS) [12,13,14] are available for the determination of rhodium in environmental samples. CSV, though it is highly sensitive, it suffers from organic matter interference. NAA could hardly be considered a routine approach because it is both time consuming and laborious. ICP-MS is a powerful technique for the determination of ultratrace amounts of rhodium, but it is very expensive to operate and a sophisticated method. GFAAS is an efficient method of determination of rhodium for its appropinquity sensitivity with ICP-MS and lower cost. Furthermore, the concentration of rhodium in environmental samples is very low, so separation and pre-concentration steps are often needed prior to analysis. Widely used techniques for separation and pre-concentration of trace amounts of rhodium include liquid-liquid extraction [15,16], solid–liquid extraction [12,17,18], and techniques based on ion exchange [3,6,19]. Cloud point extraction (CPE), which is an alternative to conventional solvent extraction, has been extensively used in analytical chemistry in the last decade [20,21]. CPE, as a new separation and pre-concentration technique, offers several advantages, such as simple operation, convenience, low cost, safety, and higher extraction and pre-concentration factors [22]. CPE is also in agreement with the principles of “Green Chemistry”, because it does not use toxic organic solvents.

**Scheme 1:**
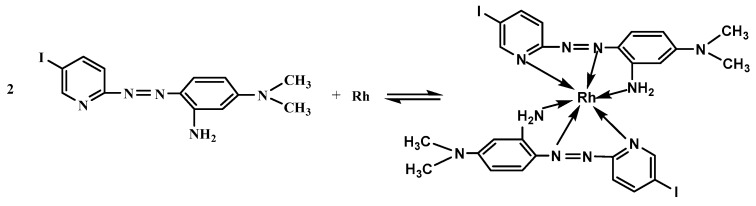
The structure of 5-I-PADMA and its rhodium complex.

The aim of this study was to combine CPE, as a highly efficient separation and pre-concentration approach, with GFAAS, as a highly sensitive detection technique, and develop a new simple method for the determination of trace amounts of rhodium in water samples. In the developed system, a newly synthesized reagent, 2-(5-iodo-2-pyridylazo)-5-dimethylaminoaniline (5-I-PADMA), which forms a hydrophobic complex compound with Rh(III) and is proved to be a highly sensitive chromogenic reagent for rhodium (ε^613^ = 1.86 × 10^5^ L·mol^−1^·cm^−1^) [23], was used as the chelating agent. The structures of the reagent and its rhodium complex are shown in Scheme 1. Triton X-114 was used as a nonionic surfactant. The main factors affecting the CPE were evaluated and optimized. The method has been successfully applied to the determination of trace rhodium in water samples. 

## 2. Result and Discussion

### 2.1. Effect of pH

**Figure 1:**
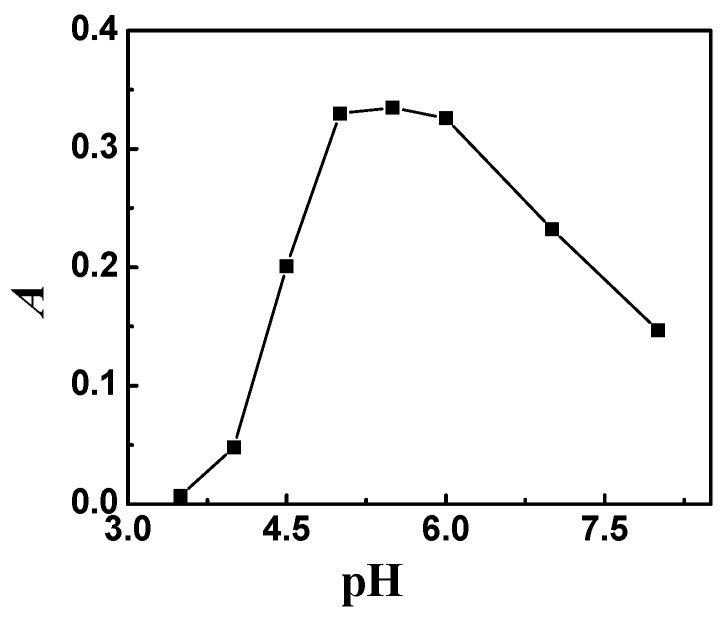
Effect of pH on absorbance. 8.0 × 10^−6^ mol/L 5-I-PADMA; 0.080% (*m*/*v*) Triton X-114; temperature: 60 °C; heating time: 15 min; 2.5 ng/mL Rh.

As shown in Scheme 1, the chelating agent 5-I-PADMA contains three nitrogen atoms (the ring nitrogen atom and the two amino group nitrogen atoms) all of which can be protonated. It can therefore exist in the solution in four states: R, HR^+^, H_2_R^2+^, and H_3_R^+^. The concentration distribution of the four species in solution is determined by its acidity. Therefore, the pH of the solution affects the formation of the chelate as well as its hydrophobicity, and is a critical parameter. According to the procedure, the influence of pH on the CPE was evaluated by changing the pH of HAc-NaAc buffer solution added in the range of 3.5–8.0. Figure 1 shows the effect of pH on extraction of rhodium chelate. It can be seen that maximum absorbance value was achieved at pH range of 5.0–6.0. Hence, a pH of 5.5 was selected for subsequent experiments.

### 2.2. Effect of the Amount of Chelating Agent

**Figure 2:**
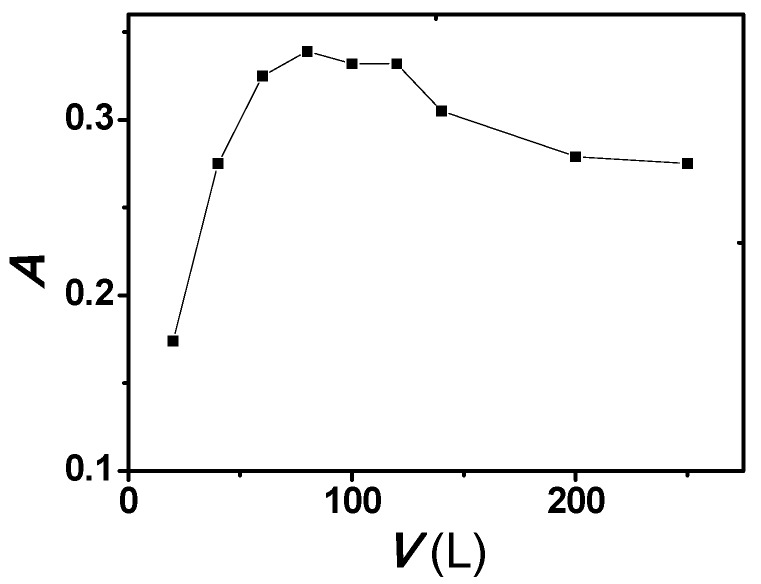
Effect of the amount of 5-I-PADAM on cloud point extraction of Rh. 0.08% (*m*/*v*) Triton X-114; temperature: 60 °C; heating time: 15 min; pH = 5.5; 2.5 ng/mL Rh.

5-I-PADMA is employed to form a hydrophobic complex with rhodium. According to the procedure, the effect of the amount of 1 × 10^−3^ mol/L 5-I-PADMA on absorbance was investigated by changing the volumes of the 5-I-PADMA added from 20 to 250 µL. As shown in Figure 2, the absorbance increased significantly with the increasing amounts of 5-I-PADMA added and reached a constant value within the range of 60–120 µL of 5-I-PADMA. However, the absorbance decreased gradually when the amount of 5-I-PADMA was beyond 120 µL. As 5-I-PADMA also had strong hydrophobicity, there was more 5-I-PADMA and less complex in the surfactant-rich phase with the further increasing of amounts of 5-I-PADMA. Hence, 80 µL 1 × 10^−3^ mol/L 5-I-PADMA was used for the subsequent experiments.

### 2.3. Effect of Triton X-114 Concentration

**Figure 3:**
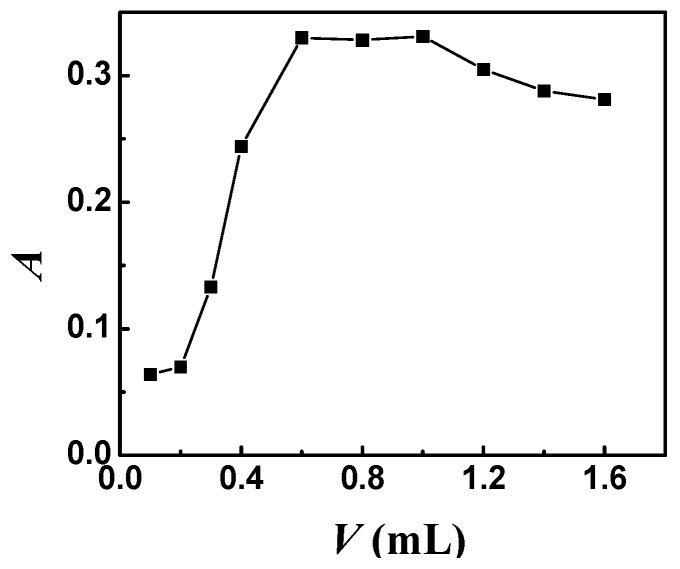
Effect of the amount of Trition X-114 on cloud point extraction of Rh. 8.0 × 10^−6^ mol/L 5-I-PADMA; temperature: 60 °C; heating time: 15 min; pH = 5.5; 2.5 ng/mL Rh.

The nonionic surfactant Triton X-114 was used as extractant because of its advantages such as commercial availability with high purity, low toxicity and cost, and relatively low cloud point temperature (23–26 °C). The concentrition of Triton X-114 not only affected extraction efficiency but also the sensitivity of the cloud point extraction. The effect of the amount of 1% (*m*/*v*) Triton X-114 on the absorbance was investigated. As shown in Figure 3, the absorbance of the rhodium chelate significantly increases when the volume of Triton X-114 increases from 0.1 to 0.6 mL. The optimal amount of Triton X-114 ranges from 0.6 to 1.0 mL. However, further increase in the volume of Triton X-114 leads to gradual decease in the absorbance signal because of the increment in the volume of the remaining phase. Therefore, 0.8 mL of 1% (*m*/*v*) Triton X-114 was employed.

### 2.4. Effect of Equilibration Temperature and Time

**Figure 4:**
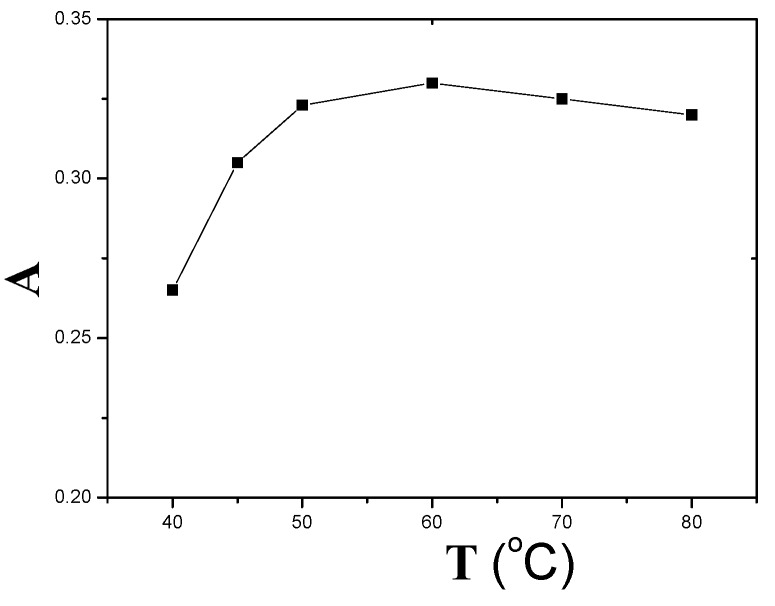
Effect of equilibration temperature on cloud point extraction of Rh. 8.0 × 10^−6^ mol/L 5-I-PADMA; 0.08% (*m*/*v*) Triton X-114;heating time: 15 min; pH = 5.5; 2.5 ng/mL Rh.

**Figure 5:**
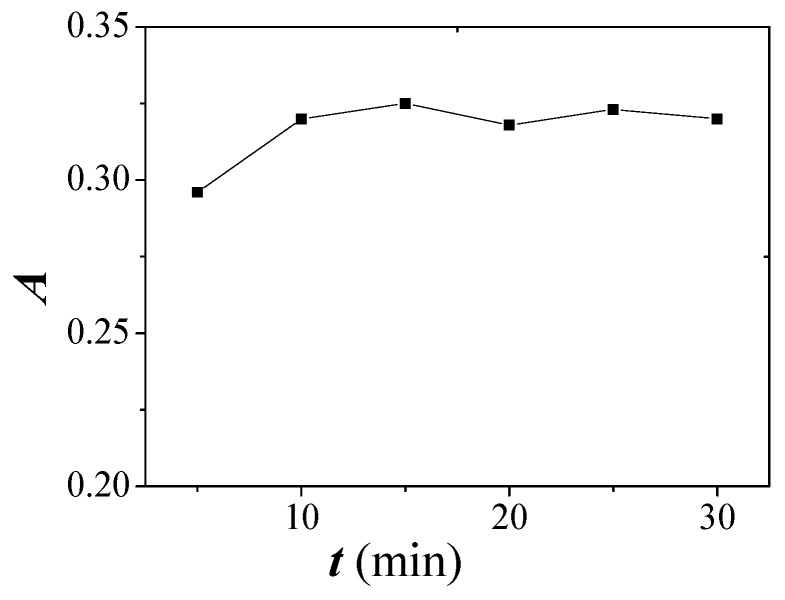
Effect of equilibration time on cloud point extraction of Rh, 8.0 × 10^−6^ mol/L 5-I-PADMA; 0.08% (*m*/*v*) Triton X-114; temperature; 60 °C; pH = 5.5; 2.5 ng/mL Rh.

Equilibration temperature and time are two important and highly effective parameters in CPE. It has been suggested that CPE processes based on the temperature-driven phase separation typical of nonionic micellar solutions should be conducted at temperatures well above the cloud point temperature of the system [24]. Thus, the effect of equilibration temperature was investigated within the range of 40–80 °C according to the procedure. The results are shown in Figure 4. It was found that absorbance increased with increase in equilibration temperature from 40 to 50 °C, and reach maximum in the range of 50–80 °C. The effect of equilibrium time was also studied in time interval of 5–30 min (Figure 5). It has been observed that when equilibration time is over 10 min, the quantitative extraction is achieved. Therefore, 60 °C and 15 min were selected as equilibrium temperature and time, respectively.

### 2.5. Interference Studies

Under the optimized conditions identified in Section 2.1 to Section 2.4, the influence of various interfering species on the determination of 1.0 ng/mL of rhodium was studied. A foreign species was considered to cause no interference when it caused a variation less than ±5% in the absorbance of the sample. The tolerance limits of various species were shown in Table 1. The results indicated that the common coexisting species did not have a significant effect on the determination of rhodium, and the developed method has very good selectivity.

### 2.6. Calibration Curve, Detection Limit and Precision

**Figure 6:**
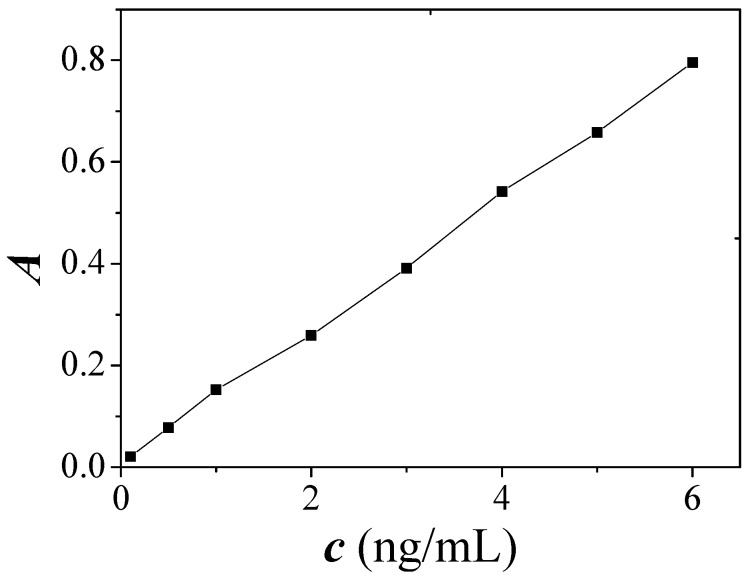
Calibration graph for Rh.

Under the optimized conditions, a calibration curve was obtained by pre-concentrating standard solutions according to the given procedure (Figure 6). The linear equation was *A* = 0.00998 + 0.1304*c* (*c*: ng/mL), with a correlation coefficient *R* = 0.9989. The calibration line was linear within the rhodium concentration range of 0.1–6.0 ng/mL. The limit of detection for rhodium, calculated as three times the standard deviation of the blank signals (3*s*), was determined to be 0.023 ng/mL. The relative standard deviation (RSD) was 3.7% (for *c* = 1 ng/mL, *n* = 11). The enrichment factor, defined as the ratio of the aqueous solution volume (10 mL) to that of the surfactant rich phase volume after dilution with HNO_3_-methanol solution (0.05 mL), was 200.

A comparison of the present method with previously reported methods involving rhodium determination in the literature in terms of detection limits and instruments employed is given in Table 2. The detection limit of the described method is superior to those given in the table, with the exception of CPE-ICP/MS. However, ICP/MS, as a multi-elemental analytical technique, is not available in many laboratories due to the high costs, while GFAAS has the advantages of simplicity, high sensitivity and low cost.

## 3. Application of Real Water Samples

In order to validate the proposed methodology, the optimized procedure was applied to the determination of rhodium in tap water, well water, spring water and river water samples. Its accuracy was checked by spiking known different concentrations of cobalt into the samples and calculating the recoveries defined as the ratio of measured amount of rhodium to the adding amount of rhodium. The results are shown in Table 3. As shown, the recoveries for the spiked samples were in the range of 96.6%~104.0%, and relative standard deviation was below 5%.

## 4. Experimental Section

### 4.1. Apparatus

Atomic absorption measurements were performed with a model TAS-990 atomic absorption spectrophotometer (Beijing Purkinje General Instrument Co. Ltd, Beijing, China) equipped with a GFH-990 graphite furnace atomizer system (Beijing Purkinje General Instrument Co. Ltd, Beijing, China). A rhodium hollow cathode lamp (Heraeus Noblelight Ltd, Shenyang, Shandong, China) was used as the radiation source. Argon was used as the purge and protective gas. The work condition of graphite furnace atomic absorption spectrophotometer was listed in Table 4. The pH values were measured by a model PB-10 pH meter (Sartorius Scientific Instrument Co. Ltd, Beijing, China) furnished with a combined electrode. A model HH-2 thermostatic bath (Kewei Yongxing Instrument Co. Ltd, Beijing, China), maintained at the desired temperature, was used for cloud point temperature experiments. A model 800-1 centrifuge (Pudong Physical Instruments Factory, Shanghai, China) was utilized to accelerate the phase separation process.

### 4.2. Reagents and Solutions

A standard stock solution of rhodium (1000 µg/mL) was prepared by dissolving spectroscopically pure (NH_4_)_2_RhCl_5_∙H_2_O in 1 mol/L HCl. Working solutions were prepared by appropriate dilution of the stock solution with water. A 1.0 × 10^−3^ mol/L 5-I-PADMA (Laboratory-synthesized [23]) solution was prepared by dissolving appropriate amounts of this reagent in ethanol. The solution of nonionic surfactant (1% *m*/*v*) Triton X-114 (Sigma-Aldrich, Milwaukee, Wisc., USA) solution was prepared by dissolving 1.0 g of surfactant in 100 mL of water. A buffer solution of pH 5.5 was prepared by 0.2 mol/L HAc and 0.2 mol/L NaAc, corrected by using a pH meter. The 0.1 mol/L HNO_3_-methanol solution was prepared by mixing of 0.2 mol/L HNO_3_ and methanol in equal volumes. All the chemicals used were of analytical reagent grade unless otherwise mentioned. All solutions were prepared with ultrapure water (18.2 MΩ cm) obtained from a Simplicity 185 (Millipore Company, Billerica, MA, USA) water purification system.

### 4.3. Procedure

An aliquot of the sample or working standard solution containing rhodium in the range of 0.1–6.0 ng/mL, 2 mL of pH 5.5 HAc-NaAc buffer solution and 80 μL of 1.0 × 10^−3^ mol/L 5-I-PADMA chelating solution were placed in a 10 mL graduated conical centrifuge tube. For the formation of rhodium complex, the mixture was heated in boiling water bath for 10 min. Afterwords for cloud point extraction, 0.8 mL of 1% (*m*/*v*) Triton X-114 solution was added and the mixture was diluted to 10 mL with ultrawater. The resultant solution was shaken and equilibrated at 60 °C for 15 min in a thermostated bath. Separation of the two phases was then accomplished by centrifugation for 5 min at 3500 rpm. The bulk aqueous phase was easily discarded by inverting the tube. The surfactant phase (the remaining phase) in the tube was heated in water bath at 100 °C to remove the remaining water, and then dissolved with 50 µL 0.1 mol/L HNO_3_-methanol solution. Then, 10 µL samples were injected into the graphite tube for GFAAS determination of rhodium. 

## 5. Conclusions

By combination of CPE as a technique for the separation and pre-concentration, with GFAAS as a detection method, we proposed a novel and sensitive method for the determination of rhodium. GFAAS is a highly sensitive analytical technique. CPE is a powerful technique for pre-concentration and separation of metal ions and it has many advantages including low cost, convenience, simplicity, safety, low toxicity and high extraction efficiency. Triton X-114 was selected for the formation of the surfactant-rich phase because of its convenient cloud point temperature and high density of the surfactant-rich phase which facilitates phase separation by centrifugation. 5-I-PADMA was chosen as the chelating reagent because it is a new and good chromogenic reagent for rhodium. The described method has been successfully applied to the selective determination of rhodium at trace levels.

## Figures and Tables

**Table 1:** Effect of foreign species on the pre-concentration/determination of rhodium.

Species	Foreign Species to Rh Ratio (*w*/*w*)	Species	Foreign Species to Rh Ratio (*w*/*w*)
Li^+^, Na^+^, K^+^, Mg^2+^, Ca^2+^, Sr^2+^, Zn^2+^, F^−^, Cl^−^, Br^−^, NO_3_^−^	2500	Cu^2+^, Ni^2+^, W(VI)	500
Ba^2+^, I^−^, SO_4_^2−^	2000	Ag^+^, Pt(IV), Ir(IV), Ru(III), Fe^3+^	200
Cd^2+^, Cr^3−^, As(V)	1500	Co^2+^	100
Pb^2+^	1000	Pd^2+^	80
Mn^2+^, Al^3+^, Mo(IV)	800		

**Table 2:** Comparison of the proposed method with reported methods using CPE prior to rhodium determination.

Reagent	Detection System	Surfactant	Limit of Detection (ng/mL)	Reference
5-(4′-Nitro-2′,6′-dichlorophenylazo)-6-hydroxypyrimidine-2,4-dione	SP	Triton X-114	0.15	[25]
-	FAAS	Span 80	1.2	[26]
2-propylpiperidine-1-carbodithioate	FAAS	Triton X-114	0.052	[27]
*O*,*O*-Diethydithiophosphate	ICP/MS	Triton X-114	0.003	[28]
2-Mercaptobenzothiazole	ICP/MS	Triton X-100	0.001	[29]
1-(2-Pyridylazo)-2-naphthol	TLS	Triton X-114	0.06	[30]
2-(5-Iodo-2-pyridylazo)-5-dimethylaminoaniline	GFAAS	Triton X-114	0.023	This work

**Table 3:** Determination results of rhodium in the water samples.

Sample	Added (ng/mL)	Found (ng/mL)	Average (ng/mL)	Recovery (％)	RSD (％)
Tap water	-	ND	-	-	-
	1.0	0.950, 0.960, 0.930 1.02, 0.970, 1.01	0.973	97.3	3.6
	3.0	3.22, 3.10, 2.94 2.96, 3.16, 3.22	3.10	103.3	3.9
Reservoir water	-	ND	-	-	-
	2.0	1.94, 2.05, 2.08 1.98, 1.94, 2.08	2.01	101	3.3
	4.0	3.92, 4.06, 3.90 3.92, 3.86, 4.07	3.96	98.9	2.2
Well water	-	ND	-	-	-
	1.0	0.970, 0.980, 1.00 0.930, 0.960, 1.04	0.980	98.0	2.9
	3.0	3.02, 2.86, 2.90 2.82, 2.88, 2.92	2.90	96.7	2.4
River water	-	ND	-	-	-
	2.0	1.96, 1.95, 2.08 1.92, 2.06, 1.92	1.98	99.0	3.6
	4.0	3.88, 3.80, 3.88 4.06, 3.92, 3.98	3.92	98.0	2.3

ND: Not detected. Tap water: *c(*Mn^2+^) = 10 ng mL^−1^, *c*(Fe^3+^) = 30 ng mL^−1^, *c*(Zn^2+^) < 50 ng mL^−1^, *c*(Cd^2+^) < 50 ng mL^−1^, *c*(Cu^2+^) < 50 ng mL^−1^, *c*(Pb^2+^) < 200 ng mL^−1^. Well water：*c(*Mn^2+^) = 10 ng mL^−1^, *c*(Zn^2+^) < 50 ng mL^−1^, *c*(Cd^2+^) < 50 ng mL^−1^, *c*(Cu^2+^) < 50 ng mL^−1^, *c*(Fe^3+^) < 30 ng mL^−1^, *c*(Pb^2+^) < 200 ng mL^−1^. Reservoir water：*c*(Fe^3+^) = 30 ng mL^−1^, *c*(Cu^2+^) = 50 ng mL^−1^, *c(*Mn^2+^) = 230 ng mL^−1^, *c*(Zn^2+^) < 50 ng mL^−1^, *c*(Cd^2+^) < 50 ng mL^−1^, *c*(Pb^2+^) < 200 ng mL^−1^. River water： *c*(Fe^3+^) = 30 ng mL^−1^, *c*(Cu^2+^) = 50 ng mL^−1^, *c(*Mn^2+^) = 230 ng mL^−1^, *c*(Zn^2+^) < 50 ng mL^−1^, *c*(Cd^2+^) < 50 ng mL^−1^, *c*(Pb^2+^) < 200 ng mL^−1^. All the water samples and their analytical results were provided by Xi׳an Hydrographic Bureau, Xi׳an, Shaanxi Province, China.

**Table 4:** Operating conditions for GFAAS.

Lamp current	6.0 mA
Wavelength	343.5 nm
Slit	0.2 nm
Filter coefficient	0.10
Pressure (Ar)	0.60 Mpa
Injected volume	10.0 μL
Drying temp.	120 °C (Ramp 5 s, hold 10 s)
Ashing temp.	1100 °C (Ramp 10 s, hold 15 s)
Atomization temp.	2300 °C (Ramp 0 s, hold 3 s)
Cleaning temp.	2500 °C (Ramp 1 s, hold 3 s)
